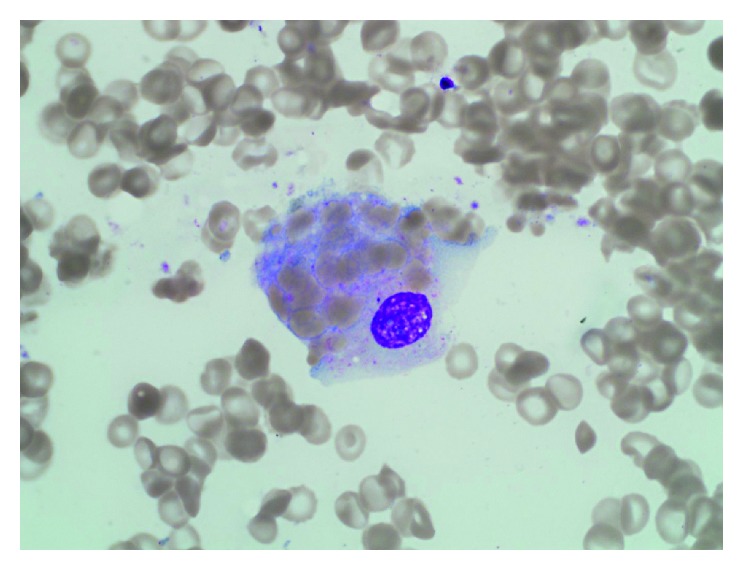# Secondary HLH Case Report Highlighting Clinical Challenges

**DOI:** 10.1155/2018/1913938

**Published:** 2018-03-04

**Authors:** Riad El Fakih, Said Y. Mohamed, Randa Alnounou, Ghada Elgohary

**Affiliations:** King Faisal Specialist Hospital and Research Center, P.O. Box 3354, Riyadh 11211, Saudi Arabia

## Abstract

A 19-year-old patient with relapsed acute myeloid leukemia (AML) developed severe and prolonged cytopenia and unexplained jaundice and fever after salvage chemotherapy. His workup revealed hemophagocytosis on the bone marrow biopsy. He was treated for HLH (hemophagocytic lymphohistiocytosis) secondary to AML and chemotherapy. The patient died on day 56 after starting his salvage chemotherapy. Unexpectedly, after his death, the microbiology laboratory reported positive mycobacterial growth from a bronchoalveolar lavage (BAL) sample taken during the workup of his fever. This case illustrates the difficulties in the diagnostic workup of HLH to identify triggers in a timely manner so that a targeted and specific therapy can be administered quickly, given the rapid and deadly evolution of the HLH process.

## 1. Introduction

**Figure 1:**
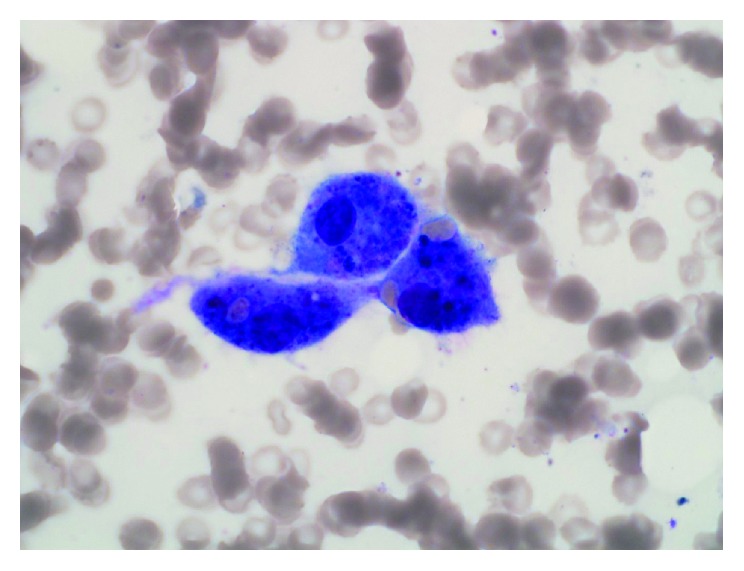


A 19-year-old patient with relapsed AML two years after his initial diagnosis was started on salvage chemotherapy (fludarabine and cytarabine). His course was complicated by unexplained jaundice and fever along with prolonged severe cytopenias. Blood and urine cultures were repetitively negative. Bronchoalveolar lavage culture was also negative including AFB stain and culture. He failed to recover his platelets or neutrophils and remained anemic up until day 28 when bone marrow biopsy (BMB) after salvage chemotherapy showed marked hypocellularity (5%), histiocytic infiltrates (Figure 1), hemophagocytosis, and no evidence of leukemia. Mycobacterial, bacterial, and fungal cultures were negative on the BMB, and no granuloma was seen on microscopic exam of the trephine biopsy. The patient also had hepatosplenomegaly, high serum ferritin (11,000 ng/ml), elevated soluble CD25 (>5000 *μ*/ml), persistent fever, and jaundice and tested negative for the familial-HLH (Hemophagocytic Lymphohistiocytosis) panel. He was diagnosed as secondary HLH due to leukemia or chemotherapy or both. He was started on HLH therapy with etoposide, IVIG, and dexamethasone with initial, yet transient and incomplete, improvement; he remained febrile, jaundiced, and pancytopenic. Liver biopsy was denied due to platelet refractoriness and abnormal coagulation panel. BMB on day 52 after chemotherapy showed persistent histiocytic infiltrates with hemophagocytosis (Figure 2) and no evidence of leukemia, and cellularity was 5%. Repeat bronchoalveolar lavage cultures and AFB stain were negative. The patient's condition deteriorated, and he died on day 56 after chemotherapy. Few days after his death, the laboratory reported positive tuberculosis growth on liquid and solid media.

## 2. Discussion

HLH is a fatal hyperinflammatory condition caused by highly stimulated but ineffective immune system. It may occur as primary HLH due to mutations in genes of the cytolytic secretory pathway, or secondary to infections, malignancy, autoimmune, or metabolic conditions. Prompt recognition is paramount, but often challenging and prognosis is poor. It is extremely rare for a patient to survive when secondary HLH is triggered by a malignant condition [1]. Despite available guidelines to help with the diagnosis [2, 3], HLH is still overlooked and often difficult to differentiate from concomitant and triggering conditions. Primary or familial HLH is diagnosed when the patient has one of the described mutations (STX11, PRF1, UNC13D, etc.) in the correct clinical setting. For secondary HLH, 8 criteria are proposed (fever, hemophagocytosis in biopsy, splenomegaly, high ferritin, elevated soluble CD25, cytopenia, low natural killer cell activity, and hypertryglyceridemia or hypofibrinogenemia) and the presence of 5/8 of these criteria confirms the diagnosis in the correct scenario [4]. Therapy is usually directed at the triggering conditions and a combination of immunomodulation and immunosuppression, which itself is risky and poses danger especially with concomitant infections. The HLH-94 protocol is a widely used regimen in adults despite the fact that it is a pediatric protocol and not studied in adults [5]. Prompt start of therapy is critical and lifesaving; however, therapy is often delayed due to delays in establishing the diagnosis. A high degree of suspicion and awareness is essential to save the lives of these patients. In our patient, we thought the trigger is AML and chemotherapy, and TB diagnosis was delayed; however, if the TB was confirmed before the patient's death, our therapy would have been changed to target both the HLH and TB. The initial but incomplete improvement of the patient to dexamethasone, IVIG, and etoposide were probably related to the partial response of the HLH process itself, but the progression and deterioration could probably be attributed to the flare of the tuberculosis infection that had been repeatedly tested negative. The lack of granuloma was probably due to severe cytopenia, effect of chemotherapy, steroids, or shadowing by HLH. Tuberculosis had been reported to complicate the use of fludarabine-based chemotherapy in many lymphoproliferative disorders including CLL and NHL [6–9], but this is the first report of TB with HLH in an AML patient after fludarabine therapy. Moreover, fludarabine and steroids have been considered as risk factors for TB activation in patients with hematological malignancies causing high mortality [10].

## 3. Conclusion

Patients with HLH are often ill and have multiple confusing conditions, which makes the diagnostic approach difficult and complicated. The case illustrates the importance that the diagnosis of HLH should be comprehensive with a high degree of suspicion with prompt confirmation via diagnostic procedures that would fulfill HLH-2004 diagnostic criteria. This should never be considered complete without looking for triggering factors. Our case illustrates the confusing and difficult diagnostic approach, the timely importance of identifying HLH and the triggering condition, the possibility of multiple triggers (AML, chemotherapy, and TB infections), and the rapid evolution of the HLH process.

## Figures and Tables

**Figure 2:**